# Microbiota and Cytokine Modulation: Innovations in Enhancing Anticancer Immunity and Personalized Cancer Therapies

**DOI:** 10.3390/biomedicines12122776

**Published:** 2024-12-06

**Authors:** Hamidreza Farhadi Rad, Hamed Tahmasebi, Samaneh Javani, Maral Hemati, Darya Zakerhamidi, Masoomeh Hosseini, Farnaz Alibabaei, Seyedeh Zahra Banihashemian, Valentyn Oksenych, Majid Eslami

**Affiliations:** 1 Student Research Committee, Jiroft University of Medical Sciences, Jiroft, Iran; 2 School of Medicine, Shahroud University of Medical Sciences, Shahroud, Iran; 3 Shahid Ashrafi Esfahani University, Esfahan, Iran; 4 Cancer Research Center, Semnan University of Medical Sciences, Semnan, Iran; 5 Faculty of Pharmacy, Tabriz University of Medical Sciences, Tabriz, Iran; 6 Department of Immunology, Semnan University of Medical Sciences, Semnan, Iran; 7 Student Research Committee, Semnan University of Medical Sciences, Semnan, Iran; 8 University of Bergen, 5020 Bergen, Norway; 9 Department of Clinical and Molecular Medicine, Norwegian University of Science and Technology (NTNU), 7028 Trondheim, Norway; 10 Department of Biosciences and Nutrition, Karolinska Institutet, 14183 Huddinge, Sweden; 11 Department of Bacteriology and Virology, Semnan University of Medical Sciences, Semnan, Iran

**Keywords:** cancer, cytokine, immunity, immunotherapy, microbiota

## Abstract

The gut microbiota plays a crucial role in modulating anticancer immunity, significantly impacting the effectiveness of various cancer therapies, including immunotherapy, chemotherapy, and radiotherapy. Its impact on the development of cancer is complex; certain bacteria, like *Fusobacterium nucleatum* and *Bacteroides fragilis*, can stimulate the growth of tumors by causing immunological evasion and inflammation, while advantageous strains, like *Faecalibaculum rodentium*, have the ability to suppress tumors by modifying immune responses. Cytokine activity and immune system regulation are intimately related. Cytokines including TGF-β, IL-6, and IL-10 promote tumor development by inhibiting efficient immune surveillance. The gut microbiome exhibits a delicate balance between pro- and anti-tumorigenic factors, as evidenced by the enhancement of anti-tumor immunity by cytokines such as IL-12 and IFN-γ. Improved immunotherapy responses are linked to a diverse microbiota, which is correlated with higher tumor infiltration and cytotoxic T-cell activation. Because microbial metabolites, especially short-chain fatty acids, affect cytokine expression and immune cell activation inside the tumor microenvironment, this link highlights the need to maintain microbial balance for optimal treatment effects. Additionally, through stimulating T-cell activation, bacteria like *Lactobacillus rhamnosus* and *Bifidobacterium bifidum* increase cytokine production and improve the efficacy of immune checkpoint inhibitors (ICIs). An option for overcoming ICI resistance is fecal microbiota transplantation (FMT), since research suggests that it improves melanoma outcomes by increasing CD8+ T-cell activation. This complex interaction provides an opportunity for novel cancer therapies by highlighting the possibility of microbiome modification as a therapeutic approach in personalized oncology approaches.

## 1. Exploring the Influence of Gut Microbiota on Anticancer Immunity

### 1.1. Cellular Aspects of Gut Microbiota on Anticancer Immunity

The cellular aspects of the gut microbiota in anticancer immunity are crucial in understanding how the microbiome modulates immune responses, particularly in the context of cancer. The ability of the body to produce a successful anticancer immune response is directly impacted by the gut microbiota, which is essential for controlling immune cell development, function, and activation. The development and function of immune cells, particularly regulatory T cells (Tregs), depend on the production of gut-associated lymphoid tissue (GALT), which it contributes to. Tregs are essential for limiting excessive immune responses that can inadvertently promote the growth of tumors because they help in the regulation of inflammation [1,2]. Certain microbial strains have a major impact on the development of naïve T cells into Tregs. For example, *Lactobacillus reuteri* increases the production of Foxp3, a Treg marker, which suppresses tumor development by limiting inflammatory pathways that may contribute to tumor progression and reduce systemic inflammation [3]. Beyond Tregs, other immune cell types involved in anticancer immunity are also influenced by the microbiome. The activation of immune cells is directly impacted by microbial strains like *Bacteroides fragilis* and *Clostridium* species. The body’s capacity to identify and react to cancers is supported by *Clostridium* species, which also improve immunological tolerance by promoting Treg differentiation. However, it has been demonstrated that Bacteroides fragilis increases the effectiveness of immunotherapies such as anti-CTLA-4 by inducing Th1 responses and activating dendritic cells via TLR2/TLR4 pathways, consequently increasing anticancer immunity even when immune checkpoint inhibitors (ICIs) are present. This highlights the critical role of the microbiota in modulating the immune response, both in maintaining health and within therapeutic contexts, where the microbiome can significantly enhance the efficacy of treatments [2,4].

The tumor microenvironment (TME), where the immune system interacts with tumor cells, is also influenced by the microbiota. Tumor-infiltrating lymphocytes (TILs), particularly CD8+ T cells and Tregs, are critical in determining the success of cancer therapies. The composition and distribution of these cells within the TME can influence patient prognosis and treatment outcomes [5]. Research has demonstrated that the gut microbiota modulates the density and spatial positioning of TILs within the TME, which affects the immune response to tumors. Studies have shown that specific microbial signatures correlate with patient responses to ICIs like anti-PD-1 therapy, suggesting that the microbiome could serve as a predictor of treatment efficacy. In this context, the microbiota not only influences immune cells directly but also alters the characteristics of the TME, potentially enhancing the tumor’s vulnerability to immunotherapies [6,7]. Further research has revealed that certain cancer treatments, such as cyclophosphamide, can cause shifts in the microbiota composition, facilitating the translocation of beneficial bacteria like *Lactobacillus johnsonii* and *Enterococcus hirae* to lymph nodes. This illustrates the dynamic and common relationship between the microbiota and immune system, where both can influence each other to enhance the overall effectiveness of cancer treatments. In general, the gut microbiota’s biological effects on anticancer immunity are complex and include alterations in immune cell activity, activation, and differentiation. The microbiome improves the effectiveness of immunotherapies and influences immune responses in the tumor microenvironment. It is important to understand these intricate relationships between immune cells and the microbiota in order to create novel therapeutic approaches that improve the effectiveness of cancer treatment by utilizing microbiome-based therapies [2,4].

### 1.2. Humoral Aspects of Gut Microbiota on Anticancer Immunity

The importance of the gut microbiota in regulating immune responses, especially in the context of anticancer immunity, has been highlighted by recent developments in cancer immunotherapy. In addition to being essential for maintaining intestinal homeostasis, the gut microbiota is also crucial for the systemic control of immunological processes, particularly through humoral processes including the synthesis of certain antibodies and metabolites. The production of secretory IgA (sIgA), which is triggered by the gut flora, is an appropriate illustration. This immunoglobulin is essential for maintaining the intestinal barrier’s integrity, preventing the entry of pathogenic microorganisms, and improving the body’s defenses against cancer. By neutralizing possible tumorigenic agents, sIgA contributes to the development of mucosal immunity, which serves as the first line of defense against carcinogenic transformations in the gut [8]. The production of metabolites, especially short-chain fatty acids (SCFAs), including butyrate, by the gut microbiota further demonstrates its impact on anticancer immunity. The capacity of SCFAs to control immune cell activities, such as promoting the activation and recruitment of immune cells like dendritic cells, neutrophils, and macrophages, is well recognized. The production of pro-inflammatory cytokines, particularly IL-6 and IL-12, is stimulated by these metabolites. These cytokines support immune surveillance and contribute to tumor suppression mechanisms. Butyrate, for instance, has been shown to inhibit cancer cell proliferation by stabilizing the gut’s epithelial layer, reducing intestinal permeability, and thereby decreasing the risk of metastasis. Through these mechanisms, SCFAs support an immunologically favorable environment that is conducive to anticancer responses, both locally and systemically [9].

Certain microbial metabolites have also been found to improve immune responses to cancer immunotherapy, in addition to SCFAs. Bacteroides pseudolongum produces inosine, which is a particularly interesting example. Along with interferon-gamma (IFN-γ), inosine stimulates the growth of Th1 cells, which are essential for the body’s reaction to immunotherapies such as CTLA-4 blockers and PD-L1 inhibitors. By influencing T cell differentiation, a key variable in determining the effectiveness of ICIs, this synergy increases their effectiveness. One promising aspect of improving the effectiveness of cancer immunotherapies and personalizing treatment plans based on a patient’s microbiome composition is the capacity of particular microbial metabolites to affect T cell responses. However, not all microbial metabolites have beneficial effects on anticancer immunity [2,4]. Some metabolites, such as polyamines, can negatively impact immune responses. Polyamines have been shown to inhibit lymphocyte proliferation and contribute to tumor invasion by imitating proteases derived from tumor cells. Similarly, imidazole propionate, a metabolite produced during dysbiosis (often associated with conditions such as type 2 diabetes), has been found to activate the mTOR signaling pathway. This pathway is crucial for regulating cell growth and metabolism, and its dysregulation has been implicated in carcinogenesis [1]. The impact of such metabolites on immune cell function and tumor progression highlights the dual nature of the microbiome in cancer immunity. Beyond the microbiome, the broader TME is integral in determining the success of cancer immunotherapies. The presence and activity of TILs, including CD8+ T cells, are essential for predicting patient prognosis. Recent studies have emphasized that the density and spatial organization of TILs within the TME significantly influence the effectiveness of ICIs. Tumor and stromal cell interactions within the TME can either enhance or hinder the immune response, underscoring the complexity of cancer immunology. Therefore, understanding the interplay between immune cell infiltration patterns and tumor cells is crucial for predicting therapeutic outcomes and optimizing immunotherapy strategies [3,10].

Diet and stress are examples of environmental variables that have been demonstrated to affect the composition of the gut microbiota and further regulate the immune response. For instance, higher responses to ICIs have been associated with certain bacterial populations, especially in individuals with metastatic melanoma. This link implies that altering the microbiota may be a practical way to enhance the effectiveness of cancer immunotherapy. Given this, combining microbiome-based therapy with conventional medications may improve the accuracy and efficacy of cancer treatments. Novel immunological surveillance techniques, including the investigation of microbial signatures, immune cell markers, and genetic alterations, have been developed as a result of the increasing understanding of the microbiome’s role in cancer immunology. By predicting and personalized immunotherapy responses, these strategies hope to open the door to more focused and efficient treatment modalities. In general, the humoral components of the gut microbiota play a critical role in determining anticancer immunity, especially its capacity to produce metabolites and modulate the immune system. The microbiome plays a crucial role in cancer immunotherapy by promoting immune responses and influencing tumor growth [11,12].

## 2. Microbial Metabolites as Mediators of Host Immune Responses in Cancer

The gut microbiota and the immune system are connected through the components and metabolic products of the microbiota. Lipopolysaccharides (LPS), found in gram-negative bacteria, activate small intestinal epithelial cells (IECs) via Toll-like receptors (TLRs), leading to the production of IL-8 by phosphorylation of IRAK and MAP. There is a significant link between LPS and TAMs. The gut microbiota employs LPS to influence the mononuclear-like macrophages (MLMs) through the LPS/TLR4 pathway, which plays a significant role in creating a precancerous inflammatory microenvironment that promotes the polarization of M1 macrophages and the secretion of pro-tumor mediators. LPS can also activate IL-17-producing T-helper cells by prompting MLMs to secrete IL-1β, which can intensify inflammation [13].

The gut microbiota, by fermentation of nondigestible polysaccharides, produces SCFAs including acetate (C2), propionate (C3), and butyrate (C4). G-protein-coupled receptors (GPCRs), particularly GPR41 (free fatty acid receptor 3; *FFAR3*), GPR43 (free fatty acid receptor 2; *FFAR2*), and GPR109A (hydroxycarboxylic acid receptor 2; *HCAR2*), are receptors of SCFAs which play important roles in the regulation of metabolism and inflammation. SCFAs have different roles, including inducing reactive oxygen species and having anti-inflammatory and antitumorigenic effects [14]. Also, SCFAs, especially butyrate, suppress the function of HDACs, leading to enhanced histone acetylation. This process results in regulating inflammatory responses and providing protection against cancer [14]. The inhibition of HDACs increases PD-1 ligand expression, enhancing immunotherapy effectiveness and inhibiting apoptosis in CD4+ T cells. This process suppresses tumor growth. The butyrate-GPR109A pathway promotes Treg and IL-10-producing T cell differentiation and inhibits NF-κB activation, a pathway active in CRC [15]. Butyrate can increase the expression of CD206, IL-4, and IL-13, regulating M2 polarization and activating the WNT-ERK1/2 signaling pathway [16]. Studies have shown that higher levels of fecal SCFAs may correlate with the effectiveness of PD-1 inhibitors in patients with solid cancer tumors, suggesting a link between gut microbiota and the effectiveness of these treatments [17]. In colon carcinoma mice treated with anti-CTLA-4 antibodies, increased butyrate and propionate concentrations in the bloodstream led to resistance, increased Treg proportion, and reduced effectiveness of anti-CTLA-4 therapy [18].

Research has indicated that in advanced glioma, levels of SCFAs decrease, while lithocholic acid (LAC) levels increase. Lithocholic acid (LCA) and deoxycholic acid (DCA) are secondary bile acids derived from the unbound forms of chenodeoxycholic acid (CDCA) and cholic acid (CA), which are produced from cholesterol in the liver. These primary bile acids are produced from cholesterol through metabolic processes in the liver. Primary bile acids are converted into secondary bile acids by the intestinal microbiota, which includes species such as *Bacteroides*, *Lactobacillus, Bifidobacterium*, and *Clostridium* (specifically clusters XIVa and XI) [19]. Ma et al. found that altering gut bacteria in liver tumor-bearing mice cause an antitumor effect associated with increased hepatic CXCR6+ NKT cells, and in human primary liver cancer tissues, higher levels of the primary bile acid CDCA were linked to CXCL16 expression, the ligand for CXCR6, and there was an inverse relationship for secondary bile acid glycolithocholate (GLCA) [20]. It has been reported that there are high fecal concentrations of bile acids in patients with CRC receiving high-fat diets [17]. By activating extracellular signal-regulated kinases (ERKs) and PKC signaling pathways and impairing the tumor suppressor p53 function, DCA may facilitate the development of CRC. DCA can trigger intestinal dysbiosis and elevate the expression of monocyte chemoattractant protein-1 (MCP-1), which is associated with the regulation of TAMs in the TME by increasing the messenger ribonucleic acid (mRNA) levels of M2 genes, ultimately leading to the polarization of M2 phenotype TAMs [21]. It also can trigger intestinal adenoma–adenocarcinoma through Wnt/β-catenin signaling pathway activation [22]. DCA has the ability to enhance the activity of cyclooxygenase-2 (COX-2) in cancer-associated fibroblasts (CAFs), and thereby can affect the TME by modifying cancer cell growth and invasion. DCA can promote the growth of colon cancer cells by enhancing the levels of COX-2, which activates epidermal growth factor receptors [23]. Also, increased levels of microbial-derived DCA can promote hepatocellular carcinoma (HCC) by provoking the senescence-associated secretory phenotype (SASP) in hepatocytes, leading to the production of pro-inflammatory and tumor-promoting factors [24].

Purine nucleosides are mainly made up of adenosine and its key metabolite, inosine, which is generated by *Akkermansia muciniphila* and *Bifidobacterium pseudolongum*. Inosine has the potential to enhance the efficacy of checkpoint blockade immunotherapy by activating antitumor T cells through its interaction with the adenosine 2A receptor (A2AR). Without glucose, inosine can serve as an alternative energy source for effector T cells, promoting their proliferation and function. In lung injury caused by LPS mice, inosine can reduce the levels of iNOS, COX2, TNF-α, IL-1β, and IL-6 by modulating the TLR4/MyD88/NF-κB signaling pathway [25].

**Figure 1:**
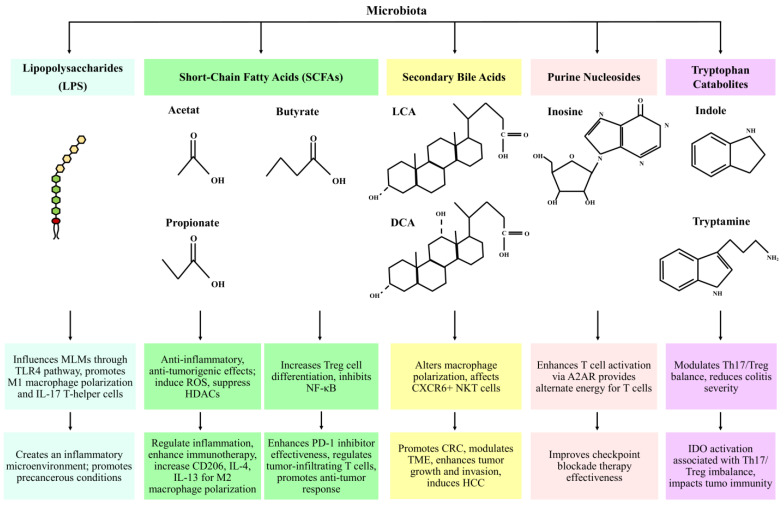
Microbiota-derived metabolites, immune interactions, and cancer-related impacts. Key pathways and bacterial contributors. MLMs: mononuclear-like macrophages. HDACs: histone deacetylases. LCA: lithocholic acid. DCA: deoxycholic acid. HCC: hepatocellular carcinoma. IDO: indoleamine 2,3-dioxygenase.

Tryptophan (TRP) is an essential amino acid that plays a critical role in immune function. Its microbial catabolites, including tryptamine, indole derivatives, and indole, contribute significantly to immune modulation. In mice with colitis, Indole-3-Carbinol has been shown to reduce Th17 cell levels while increasing Treg cell levels [15]. Mice with impaired tryptophan metabolism exhibit more severe colitis. The immune-modulatory enzyme indoleamine 2, 3-dioxygenase (IDO), which is involved in tryptophan catabolism, is activated in tumor cells. Favre et al. demonstrated that the imbalance between Th17 and Treg cells is associated with the activation of indoleamine 2,3-dioxygenase 1 (IDO1) [26]. Microbiota-derived metabolites, immune interactions, and cancer-related impacts are shown in Figure 1 and Table 1.

## 3. Immunomodulatory Roles of the Microbiome in Cancer Defense Mechanisms

The roles of *Lactobacillus* and *Bifidobacterium* in cancer immunomodulation have been increasingly recognized in recent research. *Bifidobacterium longum* and related species have demonstrated the ability to suppress the proliferation of cancer cells and improve immune surveillance, while *Lactobacillus* strains like *Lactobacillus rhamnosus* and *Lactobacillus plantarum* are known to enhance intestinal barrier integrity and modulate immune responses, potentially reducing tumor development. These genera have important anticancer properties by influencing tumor biology and interacting with the immune system. Moreover, probiotics containing these bacteria demonstrate synergistic effects when combined with immunotherapies like immune checkpoint inhibitors. For example, an enriched gut microbiome that is dominated by these strains might improve the effectiveness of therapies like PD-1 and CTLA-4 inhibition. Their potential for boosting anticancer immunity is highlighted by their impact on immune cells, such as CD8+ cytotoxic T cells, NK cells, and Tregs. These probiotics have also been shown to modulate pro-inflammatory cytokines such as IL-6 and decrease tumor volume and metastasis in experimental mice. These bacteria can increase the production of IFN-α and IFN-β by dendritic cells through the cGAS-STING signaling pathway and boost the anti-tumor performance of PD-1 immunotherapy. Also, *Lactobacillus reuteri* is able to alter CD4+ T cells into CD4 + CD8αα + double-positive intraepithelial lymphocytes that alleviate inflammatory bowel disease. *Bifidobacterium bifidum* possesses β-glucan/galactan polysaccharides on its surface, which make it capable of inducing Foxp3+ T regulatory cells and suppressing colitis. Another study demonstrated that oral intake of *Bifidobacterium* has the same impact as PD-L1 antibody, and it is effective in tumor overgrowth inhibition [1,27].

*Bifidobacterium* is an anaerobic bacterium that can grow well and sufficiently in the low-oxygen state of the tumor microenvironment, and through STING signaling and interferon type I, it can improve anti-CD47 immunotherapy, which does not allow the tumor cells to escape and causes them to be swallowed by macrophages [3]. *Streptococcus thermophiles* is another cancer-inhibiting bacterium with various functions that is majorly reduced in CRC patients; β-galactosidase, which is secreted by this bacterium, can inhibit cell proliferation and colony formation and suppress tumor growth. Interestingly, it exerts a synergic effect on other probiotics by elevating the richness of *Lactobacillus* and *Bifidobacterium*. In addition, *S. thermophiles* can also regulate lymphocytes and regulatory T-cells [1]. *Bifidobacterium pseudolongum*, *Olsenella*, and *Lactobacillus johnsonii* have been reported to boost the performance of inosine, a metabolite that can activate T helper1 cells and has a suppression effect on colon cancer and melanoma; therefore using inosine as an adjuvant can develop the efficacy of ICIs [1,28]. Programmed cell death protein 1 (PD-1)/PD-L1 and cytotoxic T lymphocyte-associated antigen 4 (CTLA-4) are inhibitory checkpoint receptors that suppress T cell activation, affecting the immune response against cancer. ICIs can arouse an immune response against cancerous cells, while *Bacteroides fragilis* activates Th1 cells, promoting PD-1/PD-L1 and CTLA-4 immunotherapy [2,9]. *Bacteroides thetaiotaomicron* and *Burkholderia cepacia* can also stimulate Th1 cells in lymph nodes and facilitate the maturation of intratumoral dendritic cells, thus enhancing the efficacy of anti-CTLA4 therapy [9]. The role of gut microbiota in modulating cancer immunity shown in Table 2.

## 4. Influence of Microbiota on Chemotherapy

The effect of microbiota on chemotherapy is divided into two parts: (Section 4.1) the effect on toxicity of chemotherapy and (Section 4.2) the effect on efficacy of chemotherapy.

### 4.1. Influence of Microbiota on Toxicity of Chemotherapy

Chemotherapy-induced toxicity has a significant relationship with the composition and functionality of the gut microbiota, impacting systemic inflammation, immune regulation, and intestinal integrity. Despite their ability to efficiently target tumor cells, chemotherapy agents frequently disturb the gut microbial ecology, which might have adverse effects. Microbial dysbiosis, which is characterized by an increase in pathogenic strains and a decrease in beneficial microbial diversity, is the result of this disturbance. These alterations have the potential to weaken the intestinal barrier and increase permeability, which promotes systemic inflammation and increases the toxicities associated with chemotherapy. Furthermore, by modulating T-cell activation, cytokine production, and other immunological pathways, the changed gut microbiota affects the host immune system and can increase inflammatory responses or impair immune homeostasis. Developing microbiota-targeted therapies, such as probiotics, prebiotics, or fecal microbiota transplantation, that reduce these side effects and enhance treatment results requires an understanding of the interaction between gut microbiota and chemotherapy-induced toxicity [29]. The most important cases that demonstrate the influence of microbiota on the toxicity of chemotherapy include the following:

(1) Oxaliplatin and microbiota-driven ROS production: Oxaliplatin relies on the production of ROS to induce apoptosis in cancer cells. However, microbiota-mediated mechanisms enhance this ROS production not only in tumor cells but also in healthy tissues. The gut microbiota modulates genes such as Nox1 and Cybb, which encode NADPH oxidase, through TLR activation. This results in an increased inflammatory cytokine profile, including IL-6 and TNF-α, contributing to systemic inflammation and exacerbation of neuropathy. This dual effect underlines the complexity of the microbiota’s involvement in chemotherapy toxicity [4]. (2) Cyclophosphamide and gut barrier disruption: Cyclophosphamide (CTX) alters the gut microbiota, increasing intestinal permeability and allowing specific bacterial species, such as *Enterococcus hirae* and *Lactobacillus johnsonii*, to translocate into the mesenteric lymph nodes and spleen. These bacteria increase inflammation and activate Th17 and memory Th1 cells, which can lead to gastrointestinal side effects such as mucositis after chemotherapy. This demonstrates the microbiota’s role in both promoting efficacy and exacerbating adverse effects [30]. (3) SCFAs and mucosal integrity: The reduction in SCFA-producing bacteria due to chemotherapy disrupts the intestinal barrier. SCFAs, especially butyrate, play a critical role in maintaining epithelial integrity and regulating inflammation. Reduced SCFA levels impair gut homeostasis, increase gut permeability, and worsen mucosal damage, heightening the risk of infections and systemic inflammatory responses during chemotherapy [31].

### 4.2. Influence of Microbiota on Efficacy of Chemotherapy

Microbiota directly impacts the efficacy of chemotherapy through its ability to metabolize drugs, modulate immune responses, and shape the tumor microenvironment. The most important cases that demonstrate the influence of microbiota on efficacy of chemotherapy include the following:

(1) Drug metabolism and activation: Certain microbiota transform chemotherapy prodrugs into their active forms, enhancing therapeutic outcomes. For example, *Lactobacillus johnsonii* enhances the immunostimulatory effects of CTX by promoting Th1 and Th17 responses. Similarly, *Barnesiella intestinihominis* augments the infiltration of γδT cells into tumor sites, boosting the immune-mediated destruction of cancer cells. These findings highlight the microbiota’s role as a metabolic intermediary critical for drug activation [30]. (2) ROS-mediated cytotoxicity in tumors: Gut microbiota support the cytotoxic effects of oxaliplatin by upregulating ROS production through immune signaling pathways. However, this effect is nuanced, as excessive ROS can also contribute to toxicity in non-tumor tissues. Balancing microbiota-mediated ROS production is key to optimizing therapeutic outcomes while minimizing side effects [32]. (3) Immune modulation by favorable microbial strains: Through the TLR2/TLR4 pathways, some bacterial species, such *Bacteroides fragilis*, increase dendritic cell activation and promote Th1 differentiation. This immune modulation amplifies the efficacy of chemotherapy by facilitating robust anti-tumor responses. Additionally, *Bifidobacterium* spp. and *Lactobacillus* spp. modulate cytokine profiles to enhance the therapeutic efficacy of chemotherapeutic agents [33]. (4) FMT and probiotics: Probiotics and FMT provide potential strategies for reducing the dysbiosis induced through chemotherapy. Beneficial strains such as *Lactobacillus* and *Bifidobacterium* recover when microbial diversity is restored with FMT. Probiotic supplements have been shown to be effective in reducing toxicities linked to chemotherapy, such as mucositis and diarrhea, while also improving treatment outcomes by increasing immune responses and preserving gut homeostasis [34].

## 5. The Role of Microbiota in Cytokine Modulation and Anticancer Immunity

The response to cancer immunotherapy dramatically varies among cancer patients. It has been demonstrated that the host immune status and diverse environment factors are closely interrelated with the clinical outcome in cancer patients. Among them, the commensal microbiota has received growing interest throughout the past decade. Recent studies have highlighted the critical role of the microbiome in the induction and progression of numerous cancers. For instance, the gut microbiota enhances the therapeutic effect of ICIs by inducing the activation and infiltration of cytotoxic T cells. Patients with more gut microbiome abundance and diversity exert better treatment outcomes in cancer immunotherapy, emphasizing the potential for microbiome regulation to improve immunotherapy efficacy. In addition to gastrointestinal microbiota, the intratumoral microbiota has also drawn increasing attention in the era of immunotherapy, since microbes colonizing the tumor microenvironment (TME) may lead to cancer progression while affecting the efficacy of cancer therapies. The frequency and composition of the tumor microbiome vary in different cancer types and depend on tumor location, host genetics, immunological state, and environmental factors. Klann et al. described that the most abundant phyla in tumor tissues of breast cancer were *Bacteroidetes*, *Firmicutes*, *Proteobacteria*, and *Actinobacteria.* Moreover, in a study by Lehr et al. [35], higher abundance of *Fusobacterium nucleatum, Clostridium colicanis,* and *Lactobacillus gasseri* or *Lactobacillus reuteri* was linked to gastric cancer. They modulate the anti-tumor immune response through several complex and multifaceted mechanisms such as direct interaction with immune cells, the production of immune suppression metabolites, production of genotoxic metabolites, molecular mimicry, and the induction of pro- or anti-inflammatory cytokines and chemokines [36].

Tumor tissues from CRC patients presented with higher concentrations of Fusobacteria, Proteobacteria, and *Ruminococcus,* which are associated with higher levels of IL-1α, IL-1β, IL-2, IL-6, IL-8, IL-9, IL-10, IL-17A, IFN-γ, TNF-α, macrophage inflammatory protein-1α (MIP-1α), macrophage chemoattractant protein-1 (MCP-1), and P-selectin [37]. However, the presence of pro-inflammatory cytokines such ad IL-17, IL-6, IL-1β, and TNF-α leads to the chronic activation of inflammatory signals that not only suppresses adaptive immune responses, but also induce tumor growth. Thereby, through the increased release of growth and immunomodulatory factors, they are correlated with poor prognosis in CRC patients [38]. Contrastingly, commensal microbiota and their products can regulate the production of cytokines with anti-tumorigenic and immunostimulatory properties, such as IL-12, as well as TNF-α and IL-22, which act as stimulators of innate immunity via pathogen-associated molecular patterns (PAMPs). IL-22 induces tissue-repairing activity and protects against infection. Moreover, *Bacteroides dorei*, *Parabacteroides distasonis*, and *Paraprevotella xylaniphila* promote IFN-γ production by CD8 T cells and subsequent anti-tumor immunity in mice [20].

Furthermore, the gut microbiota affects the innate immune cells, including neutrophils, macrophages, NK cells, and γδ T-cells. Oral treatment with *Bifidobacterium longum 51A* can increase pro-inflammatory cytokine production, such as IL-6 and TNF-α of neutrophils and their myeloperoxidase activity. It has been shown that *Bacteroides fragilis*-derived IL-17 can potentially induce MDSCs (as a hallmark of chronic inflammation) in a Th17-dependent manner to induce colon tumorigenesis in MinApc^+/−^ mice [39]. Furthermore, the microbiome residing in the TME can promote the development of dendritic cells, which further express co-stimulation/maturation markers and produce pro-inflammatory cytokines and chemokines such as TNF-α and IL-1β. Another interaction of microbiota with cytokines can be mentioned in the non-inflammatory type of tumors. For example, microbial products induce invasive colonic adenomas, leading to barrier deterioration during the tumor initiation phase, which increases the production of IL-23 and downstream cytokines like IL-17 and IL-22 that directly promote the growth of cancer cells in CRC, liver cancer [40], or pancreatic cancer. The production of IL-17 by intratumoral bacteria also facilitates B cell infiltration into tumor tissues and advances malignancy [41].

**Figure 2:**
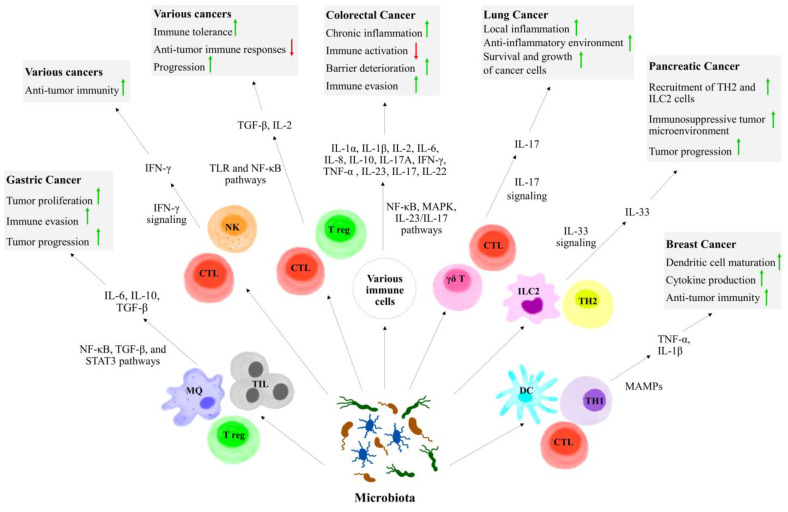
Immune cell-specific cytokines and signaling pathways in cancer progression influenced by microbiota.

The interplay between commensal microbiota, immune and tumor cells, and cytokines can determine the future of the tumor, depending on the specific context and environmental factors. For example, tumor microbiota could enhance local inflammation in lung cancer through an increase in the production of IL-17 of γδ T cells, which results in tumor progression, promoting an anti-inflammatory environment. Alam et al. found that the microbiota in pancreatic ductal adenocarcinoma tissue could increase the production of IL-33 by tumor cells, which further recruited Th2 cells and innate lymphoid cells (ILC) 2 into the tumor microenvironment, leading to tumor progression. Co-administration of IL-2 with *Akkermansia muciniphila* (AKK), an intestinal symbiont colonizing on the mucosal layer, modulates the Tregs and TGF-β levels mainly through the activation of TLR and NF-κB signaling pathways. In the other way, MAMPs in TME induce local production of pro-inflammatory cytokines (IL-1β, TNF-α, and IL-6), and continued exposure to these pro-inflammatory cytokines and MAMPs activates the NF-κB pathway, resulting in prolonged survival of the infiltrating immune cells and inducing the release of additional pro-inflammatory cytokines and oxidative factors [42]. Immune cell-specific cytokines and signaling pathways in cancer progression influenced by microbiota are shown in Figure 2.

## 6. Microbiome Profiling and Cytokine Interactions: Innovations and Applications in Personalized Cancer Therapies

It has been demonstrated that disruptions in the balance of the commensal microbiota can lead to the growth of undesirable bacteria and the manifestation of pathogenic and carcinogenic consequences; therefore, it is crucial to maintain a healthy and normal gut microbiota in order to avoid such occurrences, and to achieve proper efficacy of anti-cancer treatments. Although pathogenic microbes are responsible for the initiation and development of about 15% to 20% of cancers, commensal microbiota have a more widespread influence on the initiation and progression of tumorigenesis. According to the recent preclinical studies, human clinical studies, and meta-analyses of clinical studies, commensal microbiota can alter the host’s response to a variety of anticancer treatments, with immunomodulation emerging as one of the key mechanisms enabling these differential responses [40].

The commensal microbiota and their metabolites influence the effectiveness of cancer immunotherapy mainly through regulating both innate and adaptive immunity [43]. Immune checkpoint inhibitor (ICI) therapy is the most mature and frequent approach to cancer immunotherapy, in which the killing effect of T cells is upregulated by targeting co-inhibitory molecules including PD-1/PD-L1. *L. rhamnosus* has been demonstrated to upregulate the antitumor activity of PD-1 immunotherapy through the induction of IFN-α and IFN-β production by dendritic cells in a cGAS-STING signaling manner. *Bifidobacterium* performs anticancer activities, mainly through the initiating dendritic cell maturation, activating IFN-α and IFN-β signaling, promoting cytotoxic CD8+ T cells, and facilitating anti-PD-L1 efficacy [44].

CpG oligodeoxynucleotides (CpG ODNs) emulate bacterial DNA and induce anti-tumor effects by activating the immune system when the immunosuppressive function of IL-10 is inhibited via targeting IL-10, IL-10R, or STAT3. CpG ODNs suppress tumor proliferation by prompting tumor-infiltrating myeloid cells to release pro-inflammatory cytokines, which subsequently transition tumor-associated macrophages and dendritic cells from an anti-inflammatory to a pro-inflammatory state. A growing body of evidence suggests that impairment of the microbiota disrupts the anti-tumor effects of CpG ODNs immunotherapy, as it demonstrates that the effectiveness of CpG ODNs and anti-IL-10 treatment is diminished in germ-free and antibiotic-treated mice, which exhibit lowered levels of TNF and IL-12 compared to particular pathogen-free animals [45,46].

Not surprisingly, chemotherapy modifies the composition and diversity of microbial communities in cancerous patients, although the significance of the altered microbiome with respect to prognosis is unclear. Erlotinib, a highly specific tyrosine kinase inhibitor that can reversibly inhibit epidermal growth factor receptor mutations, is mainly used for targeted therapy following the failure of chemotherapy for non-small-cell lung cancer (NSCLC). Recently, *Bacteroides xylanisolvens* and *Bacteroides ovatus* have been shown to positively correlate with the treatment outcomes of erlotinib through upregulation of the expression of C-X-C motif ligand 9 (CXCL9) and IFN-γ in a murine lung cancer model. Moreover, butyrate, as a product of dietary fiber that fermented by gut microbiota, improved the anticancer effects of oxaliplatin via the regulation the function of CD8 + T cells in the TME through the IL-12 signaling pathway. Butyrate has also been shown to induce the production of IL-18 in intestinal epithelial cells by activating the GPR109a receptor and IL-22 production and directly affecting DCs, macrophages, and T cells. ILCs in the intestinal lamina propria generate IL-22, which stimulates the growth of epithelial cells and the synthesis of antimicrobial peptides through activating STAT3 [47].

## 7. Strategies for Enhancing Antitumor Immune Responses

Difference in host genes, separate mutations, responsible environmental factors, and the distinct composition in each person’s gut microbiota are the reasons why the responses to anticancer treatment vary from patient to patient. Since the gut microbiota’s impact in different anticancer therapies has been previously mentioned and discussed, in order to obtain an agreeable therapeutic efficacy of routine anticancer treatments, the manipulation of gut microbiota composition has been suggested [1,4]. The gut microbiota can be altered under the influence of medications, mutations, geographical location, and nutrition [2].

Dietary elements are considered as a remarkable factor that can form the gut microbiota and enrich its composition in favor of beneficial strains. For example, the use of dietary fibers has been indicated to exert anti-inflammatory effects due to the development of butyrate-producing bacteria. Butyrate is one of the SCFAs that possesses anti-inflammatory and anti-tumorigenic features [4]. Increasing the population of *Faecalibacterium prausnitzii* in order to elevate butyrate production will result in a reduction in the expression of pro-angiogenic factors and the suppression of angiogenesis [3]. By suppressing the activity of histone deacetylase and targeting the transcription factor of FOXP3, SCFAs boost the expression of regulatory T cells, which have anti-inflammatory effects on carcinogenesis. They also improve the number of macrophages and activity of CD8+ T cells [9,28]. Another dietary component is resistant starch, which can be transitioned into SCFA in the large intestine and alleviate colitis and CRC. It can both exert anti-inflammatory effects by reducing gene expression and decrease the level of COX-2, IL-1b, NF-kB, TNF-α, and anti-tumorigenic effects by increasing the expression of G-protein coupled receptor 43 or preventing histone deacetylase activity. On top of that, by inhibiting B-catenin entry to the nucleus, it can block growth factor expression and manage cell proliferation [4]. Fecal microbiota transplantation (FMT) is a route by which to manipulate the gut microbiota by transplanting the microbiome of a healthy donor in order to restore a beneficial genera and microbial diversity [2]. FMT is now FDA-approved in *C*. *difficile* infections [28]. Another manipulative agent of gut microbiota is vitamin D, which can restore its population and decrease pathogens. By mediating the expression of antimicrobial peptides, it can form phagosomes and exert antimicrobial activity. In addition, vitamin D receptors exist in various immune cells and can maintain the balance between a pro-inflammatory and anti-inflammatory state [4] Toxins are another example of microbial-derived products that normally function as virulence factors, like *Clostridium perfringens* enterotoxin, that cause food poisoning. However, they can also bind to claudin-3 and -4, tight junction proteins that are majorly expressed in human cancers, then form a pore in the plasma membrane, impair the osmotic balance between extracellular and intracellular fluids, and impose death on tumor cells. Therefore, toxins can be extracted from toxin-generating bacteria such as *Clostridium perfringens*, *Salmonella typhimurium*, and *Pseudomonas aeruginosa* and be utilized against cancer cells as chemotherapy agents; however, their virulence factors are definitely required to be modified through genetic engineering so that the potential harm to normal cells is minimized [1].

Another technique that can be employed involving the gut bacteria is the surface modification strategy, in which altering the surface of the bacteria with checkpoint blocking antibodies and tumor antigens can grant them novel biological properties that boost their anti-tumor efficacy [1]. Utilizing phages is another method of modulating the composition of gut microbiota. Using phages that are loaded with Irinotecan in order to decrease the population of *F. nucleatum*; enhance the proliferation of *C. butyricum*, which is a butyrate-producing bacteria; and suppress its role in tumorigenesis in CRCs has been an effective method to improve the efficacy of chemotherapy drugs. Other than phages, spores, the dormant bodies of fungi or bacteria, are another form of drug delivery that can bear acidic components and other chemicals in the gastrointestinal tract, release the drug directly into the tumor microenvironment, and elevate the intra-tumoral drug density, like the *C. butyricum* spore-delivering chemotherapy drug in pancreatic ductal adenocarcinoma. *Clostridium* spores can also be equipped with nontoxic prodrugs that will be later activated with gene-expressing enzymes in order to reduce the side effects of chemotherapy in normal cells [1,28].

## 8. The Gut–Immune Axis: Insights into Cancer Immunotherapy

The human immune system’s microbiota plays a critical role in controlling both innate and adaptive responses. According to research, it influences CD8+ T cells, Th1 cells, and tumor-associated myeloid cells, which helps to stimulate anticancer immune responses [48,49]. The microbiome can shape interactions between immune tumor responses and influence the immune system during carcinogenesis and cancer progression. DCs are a type of APC with different functions, including regulating T cell activation and secreting TNF as well as IFN-Is. According to Vasquez Ayala et al., the commensal bacterium *Bacteroides fragilis* triggers IFN signaling pathways, which affect DCs and strengthen Treg responses, ultimately aiding in the maintenance of intestinal immune tolerance. Additionally, research by Vétizou et al. suggests that *B. fragilis* is associated with improved responses to immune checkpoint blockade in cancer patients, likely due to its promotion of IFN signaling, as emphasized in this study [50]. Paulos et al. demonstrated that melanoma mice undergoing lymphodepletion with total-body irradiation (TBI) exhibited increased activation of DCs and elevated systemic inflammatory cytokine levels, which were mediated by TLR4 signaling [51]. In mice with melanoma and HPV E6/7-expressing lung and cervical cancers, which were undergoing radiation therapy (RT) and treated with vancomycin, Uribe-Herranz et al. found that the antitumor effects of RT were enhanced. Conversely, pretreatment with a neomycin and metronidazole regimen did not yield similar benefits. Furthermore, vancomycin treatment led to increased antigen presentation by CD11c+ dendritic cells in the tumor-draining lymph nodes of RT-treated mice, accompanied by a reduction in butyrate-producing bacteria. Given that butyrate can negatively affect APC function, it might diminish the antitumor effects of vancomycin enhanced by RT [52,53].

NKT cells are a type of T lymphocyte and are classified as unconventional T cells. While they express a TCR, the invariant or type I NKT cells specifically possess a semi-invariant TCR that interacts with glycolipid antigens (such as α-galactosylceramide (αGC)) presented by the major histocompatibility complex (MHC) class I-like protein CD1d. One key feature of these cells involves their production of a range of cytokines after a minute of activation, including IL-4 and IFN-gamma, which are generally linked to Th1 and Th2 cells, respectively, giving them anti-infectious and anti-tumor ability [54]. iNKT cells recognize various species, including *Sphingomonas*, *Ehrlichia*, *Borrelia burgdorferi*, and *Streptococcus pneumoniae*, as well as lipid antigens derived from microbes [37]. Also, B. fragilis produces glycolipids that can activate iNKT cells. iNKT cells are activated by microbiota metabolites, such as short-chain fatty acids, tryptophan metabolites, bile acids, and oxazoles [37]. Ma et al. employed a primary liver model along with three liver metastasis models to examine the effects of alterations in commensal gut bacteria, revealing a liver-specific anti-tumor response marked by an increase in hepatic CXCR6+ NKT cells. The study demonstrated that the expression of CXCL16 on liver sinusoidal endothelial cells is vital for regulating NKT cell accumulation, with primary bile acids enhancing CXCL16 levels while secondary bile acids inhibit them; additionally, treatment with the antibiotic vancomycin effectively increased NKT cell accumulation and decreased liver tumor growth [20].

Macrophages, based on their response to environmental signals, adapt their functions, shifting between two primary phenotypes: M1 and M2 [55]. M1 macrophages are pro-inflammatory cells which release cytokines such as IL-1, IL-6, and IL-23. They also express MHC II, which is stimulated by LPS, IFN-γ, and TNF-α. M1 macrophages can as anti-tumor agents by promoting Th1. In contrast, M2 macrophages possess anti-inflammatory traits, secreting cytokines like IL-10 and TGF-β. They promote the differentiation of Th2, leading to wound healing and potentially facilitating tumor progression. In the initial phases of tumor formation, the M1 phenotype predominates with an anti-tumor effect, while with tumor progression, M1 macrophages can transition into M2 macrophages, which promote tumor progression [37].

Tumor-associated macrophages (TAMs) in pancreatic ductal adenocarcinoma (PDAC) mice release cytokines and chemokines that inhibit cytotoxic T cells and promote tumor growth. When mice consume dietary tryptophan, indole-producing bacteria in their gut activate the aryl hydrocarbon receptor (AhR), reducing TNFα+IFNγ+CD8+ T cell accumulation. Inhibiting AhR reduces PDAC growth and enhances immune checkpoint blockade effectiveness. Increased levels of Fusobacterium nucleatum in CRC tissues increase TAMs, promoting CRC cell metastasis and enhancing macrophage recruitment. Upregulation of M2 markers like IL-10 and TGF-β also accelerates CRC progression [56].

## 9. Targeting the Tumor Microbiome: Implications for Immunotherapy Efficacy

Microbiota can influence both the development of tumors and their regression. A recognized risk factor for the development of gastric cancer is infection with Helicobacter pylori, which affects gastric acidity [57]. Also, by activating NF-κB and AP-1, Helicobacter pylori can increase the cancerous potential of pancreatic cells [58]. Fusobacterium nucleatum not only plays a role in the advancement and intensity of CRC, but also contributes to the metastasis of CRC, particularly in the liver [59]. Also, this bacterium has been seen in breast and pancreatic tumors [60]. PDAC patients exhibit a higher prevalence of Proteobacteria, Actinobacteria, Fusobacteria, and Verrucomicrobia compared to healthy individuals [61]. Thus, in different cancers, various microbiota can influence tumor development.

On the other hand, certain types of microbiota can enhance the efficacy of cancer immunotherapy. Immunotherapy for cancer is a strategy to fight against the disease, including approaches like immune checkpoint therapies targeting CTLA-4 and PD-1, along with adoptive T cell therapies such as chimeric antigen receptor T-cell (CAR-T) therapy [62]. CTLA-4 is an immune checkpoint protein found on T-cells. When activated, it inhibits T-cell activation by engaging the PI3K, NFκB, and MAPK signaling pathways. CTLA-4 blockade can inhibit tumor formation [63]. Ipilimumab is a monoclonal antibody that inhibits CTLA-4 and is utilized in the treatment of metastatic melanoma. Additionally, Tremelimumab is administered in combination with durvalumab for treating adult patients diagnosed with unresectable hepatocellular carcinoma (uHCC) [64].

The interaction between PD-1, a receptor on T cells, and its ligand PD-L1, present on cancer cells, sends an inhibitory signal reducing the T cells’ ability to combat cancer [65]. In ovarian cancer, the secretion of IFN-γ leads to the upregulation of PD-1 [66]. PD-1 inhibitors such as pembrolizumab, cemiplimab, and nivolumab have anti-tumor effects. A patient suffering from advanced liver carcinosarcoma underwent a combination of nivolumab and apatinib treatment, leading to partial remission of the disease [67,68]. Furthermore, Cemiplimab is FDA-approved for the treatment of metastatic or locally advanced cutaneous squamous cell carcinoma (cSCC) [69]. Routy et al. demonstrated that *Akkermansia muciniphila* enhances the effectiveness of anti-PD-L1 and anti-PD-1 therapies by facilitating the recruitment of CCR9+CXCR3+CD4+ T lymphocytes to tumor sites [70].

**Figure 3:**
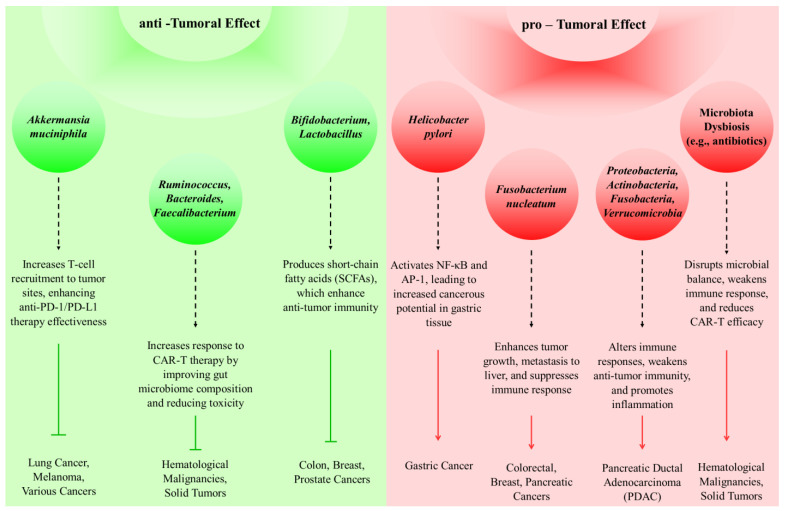
Impact of microbiome on cancer progression and inhibition: mechanisms and related cancers.

CAR-T therapy is a type of immunotherapy involving the genetic modification of T cells to express chimeric antigen receptors, enabling these T cells to have anti-tumor activity. This therapy is used in the treatment of hematological malignancies as well as solid tumors. Gut microbiota influences the efficacy of CAR-T. Smith et al. indicated that, among those with B-cell malignancies, elevated levels of *Ruminococcus*, *Bacteroides*, and *Faecalibacterium* were associated with better clinical responses. Additionally, they noted that antibiotic administration before therapy correlated with negative outcomes and heightened neurotoxicity following CAR-T therapy [71,72]. The impact of the microbiome on cancer progression and inhibition is shown in Figure 3.

The gut microbiota plays a critical role in influencing anastomotic healing following colorectal cancer surgery, primarily through its modulation of mucosal proinflammatory cytokines. Anastomotic healing is a critical determinant of postoperative recovery and long-term outcomes, and the local inflammatory response at the surgical site significantly impacts this process. The gut microbiota contributes to this response by modulating the expression and activity of key proinflammatory cytokines which are integral to tissue repair and immune system activation. Changes in the composition of the gut microbiota, known as dysbiosis, might result in different cytokine profiles in the mucosa during colorectal cancer surgery. High levels of proinflammatory cytokines, such as interleukins (e.g., IL-6, IL-1β) and tumor necrosis factor-alpha (TNF-α), may promote excessive inflammation, which may inhibit tissue regeneration and increase the risk of anastomotic leakage. Conversely, a balanced microbiome promotes collagen deposition, increased epithelial cell proliferation, and regulated inflammation, all of which are necessary for efficient recovery. According to new research, certain microbial taxa, such *Faecalibacterium prausnitzii* and *Akkermansia muciniphila*, may exert protective effects by promoting mucosal integrity and reducing inflammation. A viable approach to improving anastomotic healing and reducing postoperative complications in patients with colorectal cancer is to comprehend the interaction between the gut microbiota and mucosal cytokine dynamics [73].

The intratumoral microbiota exerts significant influence on antitumor immunity, as demonstrated by numerous studies highlighting the gut microbiota’s critical role in modulating host immune responses. These effects are bidirectional, encompassing both enhancement and suppression of antitumor immunity. Recent findings suggest that the gut microbiota facilitates improved responses to immunotherapy by modulating antitumor immune mechanisms, particularly through checkpoint blockade pathways. However, contradictory evidence indicates that the gut microbiota can also impede antitumor immunity under certain conditions. These dual roles underscore the complexity of microbiota–immune system interactions. Based on the function of the gut microbiota, new data suggest that the tumor immunological microenvironment is directly impacted by the intratumoral microbiome. The intratumoral microbiota may control the course of cancer by influencing antitumor immune responses. It seems to have two functions: either it increases immune responses and the effectiveness of immunotherapy or it reduces antitumor immunity, which promotes the growth of tumors. Understanding how microbiota-mediated pathways affect treatment outcomes requires a knowledge of the duality of these effects. For instance, specific microbial communities within tumors may bolster immune activation, improving the efficacy of immunotherapies. Conversely, other microbial populations might establish immunosuppressive niches, attenuating immune responses and promoting tumor growth. These contrasting functions highlight the need for a more thorough understanding of the processes underlying the regulatory actions of the intratumoral microbiota. Understanding these dynamics will pave the way for more targeted therapeutic interventions aimed at leveraging the microbiota’s beneficial effects while mitigating its deleterious impacts on antitumor immunity [74].

## 10. Therapeutic Modulation of Gut Microbiota in Cancer Immunotherapy: Mechanisms, Clinical Advances, and Future Directions

The human microbiome, comprising a diverse array of microorganisms and their genetic material, plays a critical role in various physiological processes, including metabolism, immunity, hormonal regulation, and overall systemic balance. The complex interaction between the gut microbiota and tumors in influencing the effectiveness of systemic anticancer treatments has been highlighted by recent studies conducted in both human and murine models. Strategies to improve treatment results by modifying the microbiota and its upstream regulatory components have been developed as a result of this link. One important factor influencing the effectiveness of cancer immunotherapy, according to recent studies, is the gut microbiota’s interaction with immune cells and cytokines in the tumor microenvironment. The gut has a significant impact on immunological responses, as evidenced by the identification of certain bacterial metabolites as essential mediators of the interaction between the microbiota and the immune system [75].

In studies involving CRC tumor-bearing mice treated with antibiotics, the gut microbiota’s composition was found to significantly influence the outcomes of anti-PD-1 immunotherapy. For example, *Akkermansia muciniphila* was associated with positive therapeutic responses, while certain bacterial compositions were correlated with reduced efficacy [76]. Antibiotic treatment was shown to disrupt glycerophospholipid metabolism, which directly affected cytokine expression in the tumor microenvironment and reduced levels of IFN-γ and IL-2, critical to effective antitumor immunity [75]. Furthermore, the administration of specific bacterial strains has demonstrated the potential to enhance the activation of CD8+ T cells, leading to improved immunotherapy responses, even in the absence of checkpoint inhibitors [75,77]. Beyond immunotherapy, the microbiome also affects the metabolism and effectiveness of chemotherapy drugs. Mice treated with antibiotics showed reduced efficacy of medications such as 5-fluorouracil and oxaliplatin, highlighting the significance of microbial enzymatic activity in preserving therapeutic potency. The anticancer activity of gemcitabine has also been linked to microbial enzymes like cytidine deaminase, which is mostly generated by Gammaproteobacteria. These data highlight the significance of the microbiota in relation to treatment regimens and show how it may affect chemoresistance [78].

FMT, a novel technique that involves transferring gut microbiota from healthy donors into patients, has shown promise in improving the results of immunotherapy. FMT in conjunction with immunotherapy has been shown to restore therapeutic responsiveness and enhance overall outcomes in notable clinical trials involving melanoma patients who were resistant to anti-PD-1 treatment. These results demonstrate how important the microbiome is for regulating immune responses and overcoming checkpoint inhibitor resistance [77]. FMT’s potential as a therapeutic approach is further supported by studies that show increased CD8+ T cell infiltration and antigen-presenting cell (APC) activation after the treatment. Variability in microbiota-related responses among cancer types is still a major challenge, despite its potential. Research shows that some bacterial families, including *Clostridiales* and *Bifidobacteriaceae*, are more prevalent among immunotherapy responders, but other bacterial families, such *Bacteroidales*, are linked to non-response. In melanoma patients on anti-PD-1 treatment, higher microbiome diversity has been associated with improved progression-free survival, highlighting the significance of microbial composition in influencing therapeutic outcomes. However, it is still unclear what processes underlie these differences and how broadly they apply to different types of cancer [79].

Future research should prioritize a deeper exploration of the gut microbiota’s systemic role in anticancer immunity. Expanding metagenomic analyses and integrating microbiota profiling into clinical decision-making frameworks could refine treatment strategies and identify biomarkers predictive of response. Personalized microbiota-based therapies, including tailored probiotics, postbiotics, dietary interventions, and optimized FMT protocols, represent promising avenues for enhancing immunotherapy efficacy. Moreover, interdisciplinary efforts are essential to unravel the complex interactions between microbiota, immune modulation, and therapeutic response, ultimately paving the way for innovative, patient-specific cancer treatments. Another important future direction is the personalization of microbiota-based therapies. Personalized strategies are essential because of the individual differences in microbiota composition and their correlation with treatment results. Individualized treatment plans may result from studies that look for microbial fingerprints that indicate how a patient will react to ICIs or chemotherapy. It is essential to have a thorough understanding of how microbiota affect immunotherapy, as well as other cancer treatments, including radiation, chemotherapy, and targeted therapies. Future studies should concentrate on the effects of microbiota on drug toxicity, resistance, and metabolism. We can create combinatorial techniques that increase the effectiveness of current treatments while reducing adverse effects by investigating these relationships.

## 11. Conclusions

In conclusion, cytokine modulation and gut microbiota have a substantial interaction that is essential for boosting anticancer immunity and increasing treatment results. The efficiency of cancer treatments, especially immunotherapy, is influenced by the gut microbiota’s crucial role in controlling cytokine patterns. Certain microbial strains have the ability to either stimulate or suppress the synthesis of important cytokines that are essential to the immune response to malignancies, including IL-6, IL-10, and IFN-γ. It has been demonstrated that beneficial bacteria, such as *Lactobacillus* and *Bifidobacterium* species, increase the synthesis of anti-tumor cytokines, which increases the effectiveness of ICIs and other cancer treatments. This highlights how certain microbiome therapies, such as dietary changes and fecal microbiota transplantation, may improve cytokine responses and strengthen anticancer immunity. The potential pathway for personalized cancer treatments that use the gut microbiota to enhance immune responses is being revealed by research into the intricacies of microbiota–cytokine interactions. By focusing on the control of cytokines through microbiome dynamics, this important area of medicine may create techniques that enhance the efficacy of cancer treatments while also improving patient outcomes in general.

## Figures and Tables

**Table 1:** Influence of gut microbiota and microbial metabolites on cancer progression and anticancer immunity.

Bacterial Species	Anticancer Immunity	Mechanism of Action	Notes	References
*Bacteroides fragilis*	↑ CRC	↑ ROS in colon epithelial cells, activates NF-κB through IL-1 and TNF-α, driving chronic inflammation and carcinogenesis	Linked with higher polyp and dysplasia rates; key risk factor in CRC	[4,9]
*Fusobacterium nucleatum*	Inhibits anticancer immunity, ↑tumor progression	Blocks NK cell activity, inhibits adaptive immune responses to evade anticancer immunity	Causes chronic inflammation and DNA damage	[1,9]
*Faecalibaculum rodentium*	↓ Tumor cell proliferation	Produces SCFAs that inhibit calcineurin/NFATc3, ↓ tumor cell growth	↑Gut homeostasis and having tumor-suppressing effects	
*Lactobacillus johnsonii*	↑Anticancer immunity, restores mucosal integrity	Translocates to lymph nodes, induces Th1/Th17 responses with IL-17 and IFN-γ during cyclophosphamide therapy	↑ Anticancer drug response by supporting T-cell activity	[2,4]
*Enterococcus hirae*	↑ Anticancer responses	Activates immune cells, especially Th1 cells	Important in chemotherapy, especially cyclophosphamide treatment	
*Bacteroides fragilis*	Essential for anti-CTLA-4 immunotherapy efficacy	Engages TLR2/TLR4 pathways to activate dendritic cells, stimulating Th1 responses with IFN-γ	Required for optimal immunotherapy response in anti-CTLA-4 treatments	
*Clostridium spp.*	Supports anticancer immunity	↑ Differentiation of CD4+ Tregs via IL-10, aiding immune tolerance and ↓ inflammation	Plays a significant role in maintaining immune regulation	
*Escherichia coli*	↓ Chemotherapy efficacy, ↑ infection risk	↑ Pathogenic strains due to altered gut microbiota, leading to epithelial barrier disruption and systemic infections	↓ Beneficial *Lactobacillus* and *Bifidobacterium* populations	
*Bifidobacterium spp.*	Protects against chemotherapy-induced toxicity, supports immune homeostasis	Contributes to barrier integrity, ↑ SCFA production which modulates IL-6 and IL-12	↓ By chemotherapy, which impacts treatment efficacy	
*B. pseudolongum*	↑ Anticancer immunity and therapeutic response	Produces inosine; with IFN-γ, ↑ Th1 differentiation, ↑ responses to anti-PD-L1 therapy	Improved outcomes in immunotherapy	[2,3]
Microbial Metabolites				
Polyamines	↓ Anticancer immunity	Inhibits lymphocyte proliferation, ↓anticancer immune responses, enables tumor protease activity	High levels in obese patients, supports tumor invasion	[9]
SCFAs	Inhibits metastasis, maintains barrier integrity, supports immune recruitment	Acts on G protein-coupled receptors, recruits/activates immune cells (neutrophils, macrophages, T-cells); modulates IL-6, IL-12	↓ Breast cancer cell metastasis, protects gut barrier integrity	
Imidazole propionate	↑ Tumor growth, especially in type 2 diabetes patients	Activates mTOR signaling, ↑ insulin resistance and tumorigenesis	Abundant in diabetic microbiota, contributing to higher cancer risk	[1]
Vitamin B (various types)	Supports metabolic functions, potentially impacting cancer development	Influences the SGOC pathway; may modulate immune responses and cellular metabolism	Impact on cancer linked to specific vitamin B pathways in SGOC metabolic regulation	
Inosine	↑ T-cell differentiation and anticancer immunity	Produced by *B. pseudolongum*, works with IFN-γ to drive Th1 cell differentiation and ↑ responses to checkpoint inhibitors like anti-PD-L1 therapy	↑ Anti-tumor responses in checkpoint inhibitor treatments	[2,3]

↑: Increase; ↓: decrease; CRC: colorectal cancer; ROS: reactive oxygen species; NK cell: natural killer cell; TLR: Toll-like receptor; SCFA: short-chain fatty acid; CTLA-4: cytotoxic T-lymphocyte associated protein 4; PD-L1: programmed death-ligand 1; mTOR: the mammalian target of rapamycin; SGOC: the serine-glycine-one-carbon.

**Table 2:** The role of gut microbiota in modulating cancer immunity and enhancing treatment efficacy.

Bacteria	Role of Signaling Pathway	Immunomodulatory Role in Cancer Defense	Refs.
*Lactobacillus rhamnosus* GG	Stimulates IFN-α and IFN-β via cGAS-STING pathway in DC	↑ Anti-tumor effects of PD-1 immunotherapy by ↑ immune response to ICIs.	[1,27]
*Lactobacillus reuteri*	Alters CD4+ T cells into CD4+ CD8αα+ intraepithelial lymphocytes	↓ IBD by modulating immune cells, contributing to overall cancer defense mechanisms.	
*Bifidobacterium bifidum*	Expresses surface polysaccharides (β-glucan/galactan) to induce Tregs	↓ Colitis and ↑ tumor inhibition, with effects similar to PD-L1 antibody therapy, by inducing Tregs.	
*Streptococcus thermophiles*	Secretes β-galactosidase enzyme	Inhibits CRC cell proliferation, ↓ tumor growth, and ↑ probiotics by regulating lymphocytes and Tregs, creating a supportive environment for immune modulation.	[1]
*Bifidobacterium* spp.	Activates STING pathway and type I interferons in hypoxic tumor microenvironment	↑ Anti-CD47 immunotherapy by ↑ macrophage-mediated phagocytosis of tumor cells, taking advantage of the tumor’s low-oxygen state.	[3]
*Bifidobacterium Pseudolongum* *Olsenella* *Lactobacillus johnsonii*	Activates Th1 cells via inosine metabolite	↑ Immune response against colon cancer and melanoma; inosine acts as an adjuvant to ↑ effectiveness of ICIs, helping ↓ tumor growth.	[1,28]
*Bacteroides fragilis*	Induces T-cell-mediated immune response by activating Th1 cells	↑ PD-1/PD-L1 and CTLA-4 immunotherapies by stimulating Th1 cells, thus ↑ anti-tumor immunity and facilitating an active immune response against cancer cells.	[2,9]
*Bacteroides thetaiotaomicron*, *Burkholderia cepacia*	Activates Th1 cells and facilitates DC maturation in lymph nodes	Boosts anti-CTLA-4 therapy efficacy by promoting maturation of DC in the tumor microenvironment and stimulating Th1 cells; ↑ immune activation against tumors.	[9]
*Gut microbiota* (General)	Upregulates Nox1 and Cybb genes for NADPH oxidase 2, aiding ROS production	Assists cytotoxic drugs (oxaliplatin) via ↑ ROS production, which induces cancer cell apoptosis, thereby ↑ chemotherapy’s anticancer effects.	[4]
*Gut microbiota* (General)	Activates TLRs to stimulate NF-κB and MAPK pathways, leading to cytokine production	Supports inflammation and ROS production in myeloid cells, ↑ immune response against cancer cells. Facilitates production of immunomodulatory cytokines to strengthen anticancer immunity alongside chemotherapy.	

↑: Increase; ↓: decrease; CRC: colorectal cancer; IBD: inflammatory bowel disease; DC: dendritic cell; MAPK: mitogen-activated protein kinases; ICIs: immune checkpoint inhibitors; ROS: reactive oxygen species; STING: stimulator of interferon genes; cGAS: cyclic GMP-AMP synthase; PD-1/PD-L1: programmed death-1/programmed death-ligand 1; NF-κB: nuclear factor kappa B.
